# *Echinococcus multilocularis* and other zoonotic helminths in red foxes (*Vulpes vulpes*) from a southern German hotspot for human alveolar echinococcosis

**DOI:** 10.1186/s13071-023-06026-2

**Published:** 2023-11-18

**Authors:** Carina Schneider, Wolfgang Kratzer, Andreas Binzberger, Patrycja Schlingeloff, Sven Baumann, Thomas Romig, Julian Schmidberger

**Affiliations:** 1 Department of Parasitology, Hohenheim University, 70599 Stuttgart, Germany; 2 Klinik Für Innere Medizin I, Universitätsklinikum Ulm, Albert-Einstein-Allee 23, 89081 Ulm, Germany

**Keywords:** *Echinococcus multilocularis*, Alveolar echinococcosis, Distribution, *Vulpes vulpes*, Intestinal helminths

## Abstract

**Background:**

We describe the spatial distribution of *Echinococcus multilocularis* in its main definitive host, the red fox, and the distribution of human cases of alveolar echinococcosis (AE) within a highly endemic focus in southern Germany (13.7–19.9/100,000 in 1992–2018). Human cases were unequally distributed within the endemicity focus. The purpose of the study was to test whether this is reflected in the small-scale distribution of *E. multilocularis* in foxes.

**Methods:**

Three areas with contrasting numbers of human cases were selected within the counties of Ravensburg and Alb-Donau, Baden-Württemberg, Germany. From 2018 to 2020, a total of 240 fox carcasses were obtained from traditional hunters in these areas. Carcasses were necropsied and examined for the presence of intestinal helminths. The statistical analysis was performed with SAS version 9.4, and the geo-mapping with QGIS version 3.16.0 Hannover.

**Results:**

The prevalence of *E. multilocularis* in foxes was 44/106 (41.5%) in area I (commune Leutkirch and environs), 30/59 (50.8%) in area II (commune Isny and environs), and 31/75 (41.3%) in area III (commune Ehingen and environs). From 1992 to 2018, a total of nine human cases of alveolar echinococcosis were recorded in area I, five cases were recorded in study area III, and no cases were recorded in area II. No statistically significant differences between the areas were observed (*P* > 0.05) for intestinal infections with *E. multilocularis*, and no apparent spatial correlation with the small-scale distribution of human cases was found. Concerning other zoonotic helminths, *Toxocara* spp. were equally common, with prevalence of 38.7%, 47.4% and 48.0%, respectively, while the frequency of *Alaria alata* varied among the study areas (0.0–9.4%), probably reflecting the specific habitat requirements for the establishment of its complex life cycle.

**Conclusions:**

*Echinococcus multilocularis* is highly prevalent in foxes in all the studied areas. The varying number of human AE cases within these areas should therefore be caused by factors other than the intensity of parasite transmission in foxes.

**Graphical Abstract:**

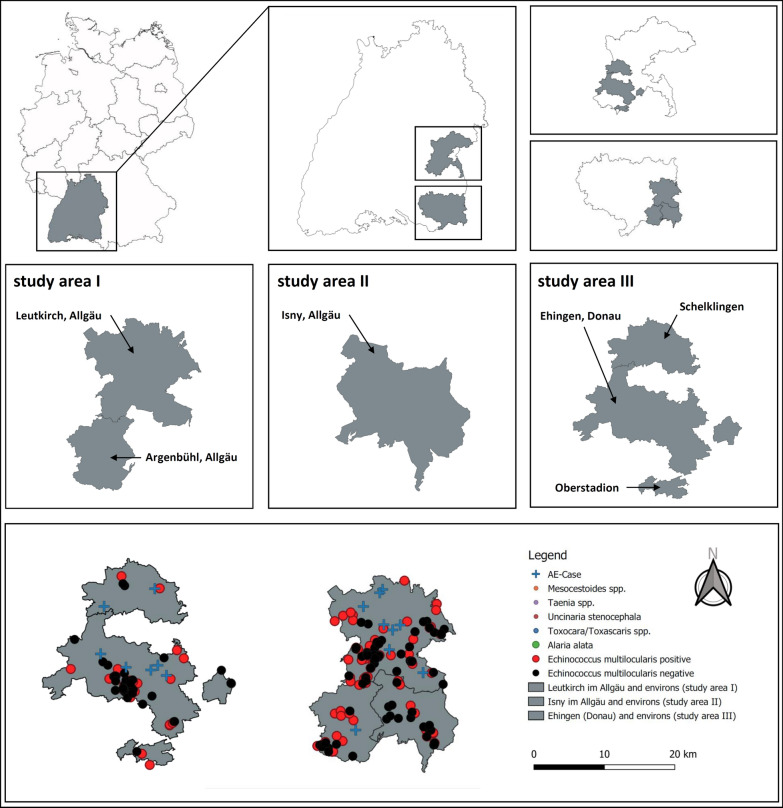

## Background

Human alveolar echinococcosis (AE) is a rare zoonotic disease, caused by the parasitic cestode *Echinococcus multilocularis* [1–3]. The parasite is exclusively present in temperate and subarctic/arctic parts of the Northern Hemisphere [1, 4]. In western-central Europe, the most affected countries are France, Germany, Switzerland and Austria [1, 4, 5].

Approximately 70–80% of human AE cases in Germany originate from the two southernmost federal states of Baden-Württemberg and Bavaria. Areas of highest infection risk are in the mountainous regions of the Alps and the Swabian Jura [6]. The most recent large-scale studies of the main definitive host, the red fox (*Vulpes vulpes*), in Baden-Württemberg and Bavaria were conducted from 1999 to 2010 and reported prevalence estimates of 37.0–55.5% *E. multilocularis* infections [7].

The life cycle of *E. multilocularis* includes predatory definitive hosts and herbivorous intermediate hosts. In Europe, definitive hosts are mostly red foxes (*V. vulpes*), occasionally raccoon dogs (*Nyctereutes procyonoides*), domestic dogs and cats, where the adult worms settle in the small intestine [8]. Parasite eggs are shed via the faeces into the environment, where they are accidentally ingested by intermediate hosts, most importantly common voles (*Microtus arvalis*) and other arvicoline rodents such as water voles (*Arvicola* spp.)*,* bank voles (*Myodes glareolus*) or muskrats (*Ondatra zibethicus*). In intermediate hosts, oncosphere larvae hatch from the eggs in the intestine, penetrate the intestinal wall and are distributed in the body via the circulatory system. The first stages of further development into metacestodes take place almost exclusively in the liver parenchyma, where they form conglomerates of microscopic cysts, eventually leading to a solid, tumour-like structure that replaces liver tissue and may also grow into other organs, even forming metastases. In suitable intermediate hosts, the infection is typically fatal after a period of several months [9]. Eventually, the metacestodes are filled with protoscoleces, which, upon predation by a definitive host, lead to a new generation of adult worms in the definitive host’s intestine.

Humans, by acting as ‘dead-end hosts’, take no part in the parasite’s life cycle: like real intermediate hosts, they ingest worm eggs through the faecal-oral route, which eventually leads to the development of infiltrative metacestodes, in 98% of cases located in the liver, causing AE [2]. In contrast to rodent intermediate hosts, the progress of parasite growth in humans can be extremely slow, and asymptomatic periods of up to 15 years after infection have been estimated [2].

While the large-scale geographical region has been well described as determining infection risk [6], there are indications that some risk factors may act on smaller scales, e.g. at the level of communes, where differences in the number of human cases have been reported. The aim of our study was to correlate the small-scale distribution of fox infection with *E. multilocularis* and other zoonotic intestinal fox helminths with the distribution of human AE cases. The study was conducted in areas where data from the National Echinococcosis Registry show high prevalence of human AE during the period 1992–2018 (counties Ravensburg and Alb-Donau [6, 10, 11]).

## Methods

### Selection of study regions

The selection of the study regions was based on the voluntary National Echinococcosis Registry Germany and the human cases of AE recorded during the period from 1992 to 2018 [6]. In the reported period, 37 human disease cases each were registered in the districts of Ravensburg (13.7/100,000 inhabitants) and Alb-Donau (19.9/100,000 inhabitants), parts of Baden-Württemberg. The small-scale distribution showed high numbers of cases, especially for the Ravensburg district, with a total of nine cases in Leutkirch im Allgäu and five cases in Ehingen (Donau). Studies on foxes have shown very high infection rates since 1988, especially in the above-mentioned regions [12].

Based on the high number of cases, Leutkirch im Allgäu and environs and Ehingen (Donau) and environs were selected as study areas. Isny im Allgäu, which is adjacent to Leutkirch im Allgäu and environs, has no known registered human cases of the disease, but was also selected as a study area.

### Data on human AE cases

The National Echinococcosis Registry Germany was established in 2016 as part of a project funded by the German Research Foundation (DFG) [6]. All cases of the disease with detailed information on epidemiology, risk factors, diagnostics, treatment and care are recorded in the registry on a voluntary basis. The disease registry closes the knowledge gap in the existing reporting obligation according to the Infection Protection Act (IfSG). The National Echinococcosis Registry Germany records cases from 1992 to the present.

### Fox sampling

The study and examination of foxes took place during the period August 2018–April 2020. During this period, foxes were shot during official hunting in the selected study regions Leutkirch im Allgäu and environs (study area I), Isny im Allgäu and environs (study area II) and Ehingen an der Donau and environs (study area III) and examined for the presence and infection level of helminths. After shooting, the foxes were stored at −20 °C and examined in a biosafety level 3 (S3) laboratory at the University of Hohenheim, Parasitology Section. Heavily decomposed foxes and foxes which were no longer able to be examined due to injuries to the intestine were excluded from the examination. In total, 27 out of 267 foxes had to be excluded for these reasons.

### Examination of foxes

The small intestines of foxes were removed from the carcasses, longitudinally opened and examined for intestinal helminths using the intestinal scraping technique (IST) [13]. Examinations were performed in a biohazard laboratory according to safety regulations.

### Statistical analysis

Statistical analysis was performed using SAS statistical software version 9.4 (SAS Institute, Cary, NC, USA). The mean, standard deviation (SD), minimum (Min), maximum (Max), and absolute and relative frequencies were calculated first as qualitative characteristics.

$$\mathrm{Prevalence}= \frac{\mathrm{number\, of\, patients\, at\, one\, time}}{\mathrm{total\, population}} \times 100,000\, \mathrm{inhabitants}$$

Prevalence per 100,000 population was calculated based on population data from the 2011 census using the above formula. Differences in frequency distributions were calculated using the Chi-square and Fisher exact test statistics. All tests were two-sided. The significance level was set at *α* = 0.05. Values of *P* < 0.05 were considered significant. Patients’ place of residence 10 years before initial diagnosis was georeferenced according to the Universal Transverse Mercator (UTM) coordinate system. Geo-mapping of the shapefiles was performed using the QGIS version 3.16.0 Hannover geographical analysis system.

## Results

### Human alveolar echinococcosis in the study areas, 1992–2018

From 1992 to 2018, a total of nine human AE cases were registered in study area I (Leutkirch im Allgäu and environs), including 5/9 (55.6%) female and 4/9 (44.4%) male, with a mean age of 54.5 ± 17.3 years at first diagnosis. The prevalence in area I during this period was 29.8 cases per 100,000 inhabitants. For 6/9 (66.7%) cases, diagnosis was accidental. Two of nine patients reported an agricultural occupation, and 7/9 (77.8%) reported ownership of dogs (Table 1). For study area II (Isny im Allgäu and environs), there were no human AE cases reported in the National Echinococcosis Registry Germany. From 1992 to 2018, a total of five human AE cases were registered for study area III (Ehingen [Donau] and environs), 2/5 (40.0%) male and 3/5 (60.0%) female, with a mean age of 55.5 ± 18.2 years at first diagnosis. The prevalence in area III during this period was 13.9 cases per 100,000 inhabitants. With 3/5 (60.0%) cases, diagnosis was accidental; 2/5 (40.0%) reported an agricultural occupation, and 3/5 (60.0%) reported ownership of dogs (Table 1).

**Table 1:** Patient characteristics of human disease cases in study areas, 1992–2018

	Leutkirch im Allgäu and environs (area I) *n* = 9 AE cases	Isny im Allgäu and environs (area II) *n* = 0 AE cases	Ehingen (Donau) and environs (area III) *n* = 5 AE cases
	*n* (%)	*n* (%)	*n* (%)
Age at initial diagnosis			
Mean ± SD (Range)	54.5 ± 17.3 (22–79)		55.0 ± 18.2 (27–78)
Sex			
Male	4 (44.4%)		2 (40.0%)
Female	5 (55.6%)		3 (60.0%)
Incidental finding	6/9 (66.7%)		3/5 (60.0%)
Dog ownership	7/9 (77.8%)		3/5 (60.0%)
Agricultural activity	2/9 (22.2%)		2/5 (40.0%)
Human population	30.181 inhabitants	14.735 inhabitants	35.870 inhabitants
Prevalence/100,000	29.8/100,000	0.0/100,000	13.9/100,000

#### *Echinococcus multilocularis* and other helminths in foxes in the study areas

From August 2018 to April 2020, a total of 240 foxes (from 267 collected carcasses) were examined from three study areas: (1) Leutkirch im Allgäu and environs (area I; *n* = 106), Isny im Allgäu and environs (area II; *n* = 59) and Ehingen an der Donau and environs (area III; *n* = 75). Total prevalence was 43.8% for *E. multilocularis*, 35.0%, for *Taenia* spp., 48.8% for *Mesocestoides* spp., 43.8% for *Toxocara*/*Toxascaris* spp., 19.2% for *Uncinaria stenocephala* and 5.4% for *Alaria alata*.

#### Area I, Leutkirch im Allgäu and environs (Ravensburg district, Baden-Württemberg)

**Fig. 1:**
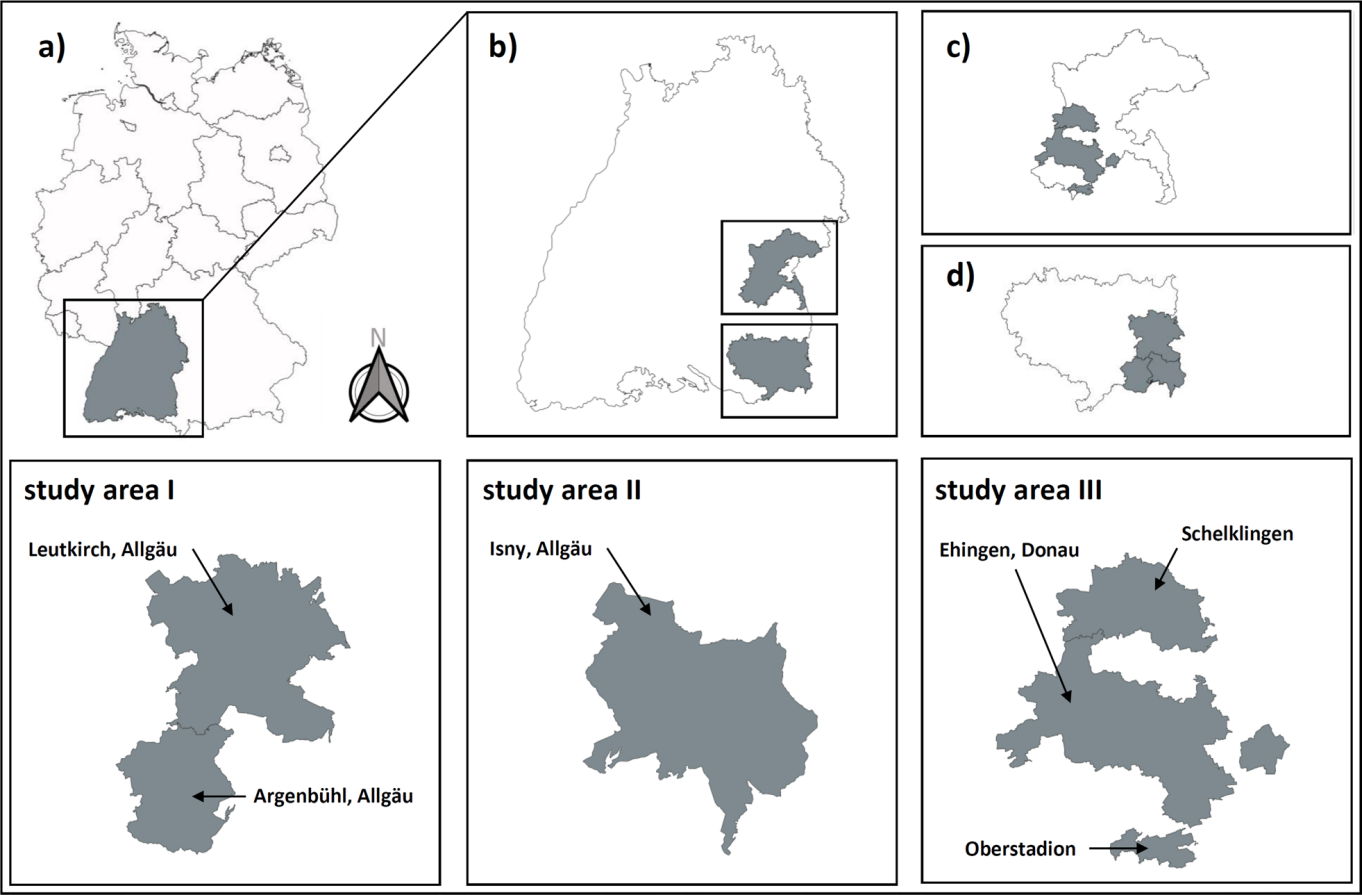
Study areas and their localization. **a** The study regions are located in Germany, Baden-Württemberg in the districts of **b** Ravensburg and Alb-Donau. **c** and **d** show the study areas Ehingen (Donau) and environs as well as Leutkirch im Allgäu and Isny im Allgäu with environs. Study area I comprises Leutkirch im Allgäu and Argenbühl. Study area II comprises Isny im Allgäu, and study area III comprises Ehingen (Donau) with Schelklingen and Oberstadion

**Fig. 2:**
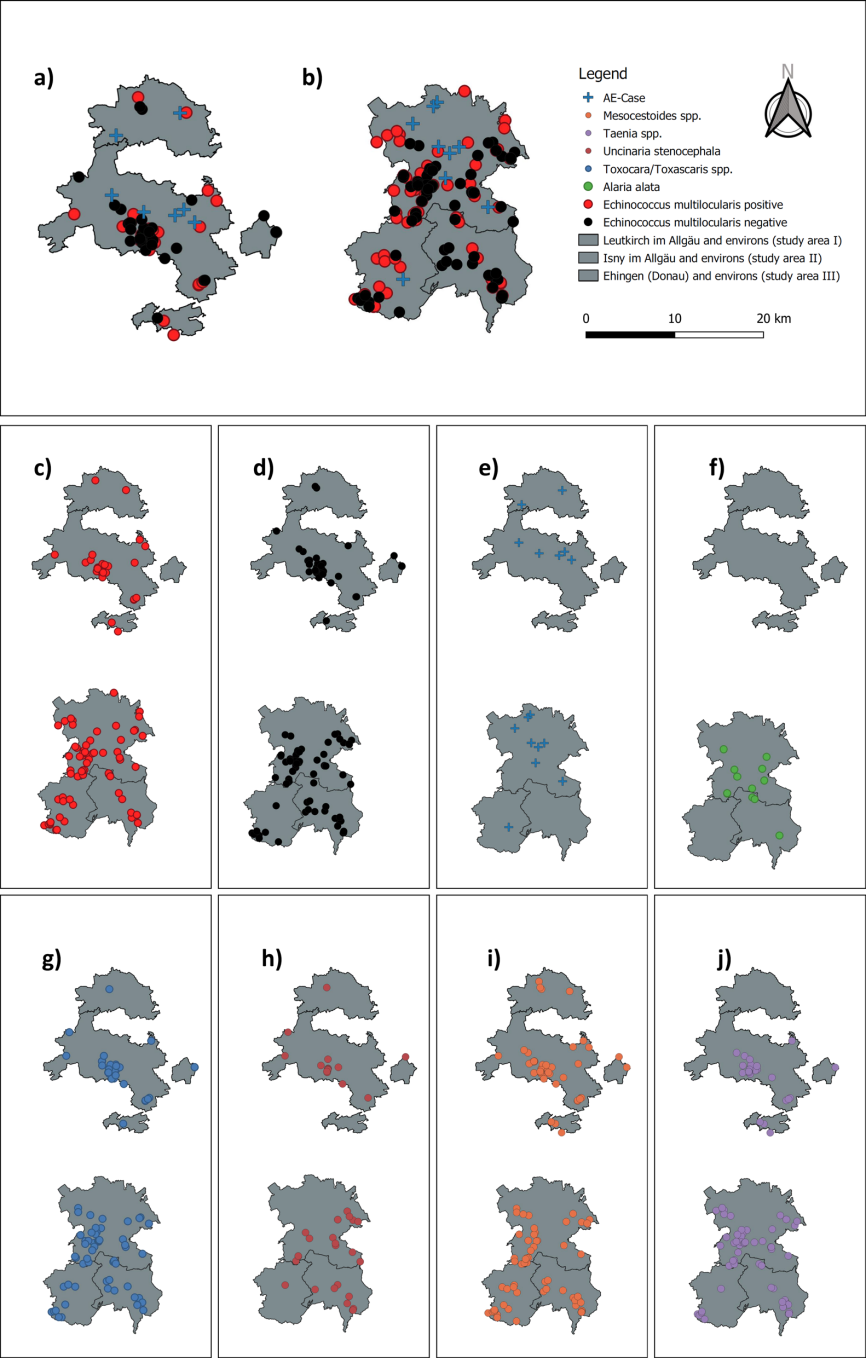
Geographical distribution of human AE cases and fox infection with *E. multilocularis* and other helminths. **a** and **b** Human AE cases (blue cross), foxes positive (red dot) and negative (black dot) for *E. multilocularis*. **a** Study area III; **b** study areas I and II, **c** foxes positive for *E. multilocularis,* **d** foxes negative for *E. multilocularis,* **e** human AE cases, **f** foxes positive for *Alaria alata*, **g** foxes positive for *Toxocara*/*Toxascaris* spp., **h** foxes positive for *Uncinaria stenocephala,* **i** foxes positive for *Mesocestoides* spp., **j** foxes positive for *Taenia* spp.

Out of 106 foxes, 11/106 (10.4%) were juvenile and 95/106 (89.6%) were adult; 48/106 (45.3%) were male and 58/106 (54.7%) were female; 44/106 (41.5%) were infected with *E. multilocularis*. Other intestinal helminths occurred at a prevalence of 10/106 (9.4%) for *A. alata*, 32/106 (30.2%) for *Mesocestoides* spp., 38/106 (35.8%) for *Taenia* spp., 41/106 (38.7%) for *Toxocara*/*Toxascaris* spp. and 19/106 (17.9%) for *U. stenocephala* (Tables 2 and 3, Figs. 1 and 2).

**Table 2:** Prevalence of *E. multilocularis* and other helminths in foxes of the three study areas

	Leutkirch im Allgäu and environs (area I) Aug 2018–Jan 2019 *n* = 106		Isny im Allgäu and environs (area II) Aug 2018–Apr 2019 *n* = 59		Ehingen (Donau) and environs (area III) Aug 2019–Apr 2020 *n* = 75	
	No. pos	Prevalence (%)	No. pos	Prevalence (%)	No. pos	Prevalence (%)
*Echinococcus multilocularis*	44	41.5	30	50.8	31	41.3
*Taenia* spp.	38	35.8	19	32.2	27	36.0
*Mesocestoides* spp.	32	30.2	33	55.9	52	69.3
*Toxocara*/*Toxascaris* spp.	41	38.7	28	47.4	36	48.0
*Uncinaria stenocephala*	19	17.9	11	18.6	16	21.3
*Alaria alata*	10	9.4	3	5.1	0	0.0

**Table 3:** *Echinococcus multilocularis* prevalence in foxes, stratified by age, sex and study areas

	Leutkirch im Allgäu and environs (area I) Aug 2018–Jan 2019 *n* = 106		Isny im Allgäu and environs (area II) Aug 2018–Apr 2019 *n* = 59		Ehingen (Donau) and environs (area III) Aug 2019–Apr 2020 *n* = 75	
	No. pos/total no.	Prevalence (%)	No. pos/total no.	Prevalence (%)	No. pos/total no.	Prevalence (%)
Age						
Juvenile	10/11	90.9	2/4	50.0	12/25	48.0
Adult	34/95	35.8	28/55	50.9	19/50	38.0
Sex						
Male	24/48	50.0	14/24	58.3	17/43	39.5
Female	20/58	34.5	16/35	45.7	14/31	45.2
Unknown	0/0	0.0	0/0	0.0	0/1	0.0

#### Area II, Isny im Allgäu and environs (Ravensburg district, Baden-Württemberg)

Out of 59 foxes, 4/59 (6.8%) were juvenile and 55/59 (93.2%) were adult; 24/59 (40.7%) were male and 35/59 (59.3%) were female; 30/59 (50.8%) were infected with *E. multilocularis*. Other intestinal helminths occurred at prevalence of 3/59 (5.1%) for *A. alata,* 19/59 (32.2%) for *Taenia* spp.*,* 28/59 (47.4%) for *Toxocara*/*Toxascaris* spp., 33/59 (55.9%) for *Mesocestoides* spp. and 11/59 (18.6%) for *U. stenocephala* (Tables 2 and 3, Figs. 1 and 2).

#### Area III, Ehingen an der Donau and environs (Alb-Donau-Kreis district, Baden-Württemberg)

Out of 75 foxes, 25/75 (33.3%) were juvenile and 50/75 (66.6%) were adult; 43/75 (57.3%) were male, 31/75 (41.3%) were female, and 1/75 (1.3%) could not be allocated to any sex due to carcass damage; 31/75 (41.3%) were infected with *E. multilocularis.* Other intestinal helminths occurred at prevalence of 0/75 (0.0%) for *A. alata,* 52/75 (69.3%) for *Mesocestoides* spp., 27/75 (36.0%) for *Taenia* spp., 36/75 (48.0%) for *Toxocara*/*Toxascaris* spp. and 16/75 (21.3%) for *U. stenocephala* (Tables 2 and 3, Figs. 1 and 2).

#### Differences between the areas

For *E. multilocularis*, no statistically significant differences in prevalence were found between areas I and II, I and III, or II and III (*P* > 0.05). The *E. multilocularis* prevalence stratified by age and sex of foxes shows differences between the study regions (Table 3).

For *Mesocestoides* spp., there were statistically significant differences in prevalence between areas I and II (32/106 vs 33/59; *χ*^2^ = 10,5212; *df* = 1, *P* = 0.0011) and between areas I and III (32/106 vs 52/75; *χ*^2^ = 27,0607; *df* = 1, *P* < 0.0001). For *A. alata*, statistically significant differences existed between areas I and III (10/106 vs 0/75; Fisher’s test: *P* = 0.0056), but not between areas II and III or areas I and II. For *Taenia* spp., *Toxocara*/*Toxascaris* spp. and *U. stenocephala,* no statistically significant differences in prevalence were found between the areas (*P* > 0.05) (Table 2).

## Discussion

Our study areas in the pre-alpine region of Allgäu and the Danube valley in Baden-Württemberg, bordering Bavaria, are well known as hotspots of human AE in central Europe [6]. Despite this, few surveys have ever been conducted there on the frequency of *E. multilocularis* or other intestinal helminths of foxes. The most recent prevalence estimates date back to 1993/1994, when 679 foxes were examined for intestinal helminths in the extensive administrative district of Tübingen, which includes our study sites but additionally covers almost a quarter of Baden-Württemberg. There, 44.8% of foxes were infected with *E. multilocularis*, 21.4% with *Taenia* spp., 15.2% with *Mesocestoides* spp., and 22.1% with *Toxocara*/*Toxascaris* spp. [14].

The focus of our study was the correlation of *E. multilocularis* infection in foxes with the presence of human AE cases on small spatial scales. A randomized epidemiological study with ultrasound from the year 2002 with 2500 inhabitants of the rural town of Leutkirch im Allgäu did not detect any cases of AE [15]. In contrast, nine AE cases were diagnosed via the regular health system in the same town and surroundings in the period 1992–2018, indicating a high infection pressure. A complete lack of human AE cases in the neighbouring town of Isny raised the question of possible small-scale factors affecting infection pressure. One such possible factor, the prevalence of *E. multilocularis* in the animal life cycle, was investigated in this study. We showed that fox infection was not statistically different among the three study areas, arguing against a direct correlation. Moreover, the positive foxes were evenly distributed across the study areas without apparent clustering. This does not argue against the existence of transmission foci where rodent infection is particularly high, but indicates that even if such foci exist, the mobility of foxes distributes the parasite over larger areas. However, it must be considered that the infection events of current human AE cases may date back as many as 15 years [2, 16], a time for which no retrospective data on fox infection are available on such small scales. Also, the registry data basis for human AE cases in our study may be skewed due to underreporting and diagnostic bias [6, 17, 18]. Other factors apart from the intensity of the wildlife cycle may also be of relevance. Urbanization of foxes, carrying the parasite life cycle into close contact with the human population [19], was not studied here and may be present at different levels in each study area. The same applies to the role of domestic dogs and cats, which may carry the parasite into peoples’ environs via intestinal infection or contamination of fur [20].

The drastic rise in fox populations after the eradication of fox rabies in the 1990s accompanied by the equally drastic increase in *E. multilocularis* prevalence in wild mammals stimulated numerous surveys of this parasite in Germany and surrounding countries during this period [21, 22]. By the early 2000s, research interest in this subject had decreased considerably, and current data are now scarce for most regions of central Europe [7]. Our study closes a gap of knowledge for at least a limited part of southern Germany.

In the following we compare prevalence data for the various helminths from our survey with recently published results from other areas. It needs to be considered that in most recent studies [23–25], fox intestines were examined using some variant of the sedimentation and counting technique (SCT) [26], while in our study we employed IST in order to keep the method identical to previous surveys in this region. IST was shown to have a reduced sensitivity of 73–78% for detection of *E. multilocularis* compared with SCT and its variants [27, 28]. No test evaluations are available for other helminths, but reduced sensitivity is likely to be similar for other small species (e.g. *U. stenocephala*, *A. alata*), but unlikely for large, macroscopically visible helminths (*Taenia* spp., *Mesocestoides* spp., *Ascarids*).

For *E. multilocularis*, the prevalence in our three study areas was very similar, indicating a homogeneous situation on a larger scale. Comparison with the above-mentioned study from 1993/1994, conducted with the same examination method [14], also shows an almost identical level of prevalence (44.8 vs 43.8%). Although the study areas overlap, but are not identical, this indicates a very stable epidemiological situation of *E. multilocularis* in the region within the past 25 years. This may not be the case for *Taenia*, *Mesocestoides* and ascarid nematodes, which were more frequent in our sample than in the previous study. This is interesting information and indicates that, despite partly identical intermediate hosts and transmission mode to foxes, the response of various rodent-transmitted helminths to environmental parameters can differ. This has also been shown during an anthelmintic deworming study of foxes in the vicinity of our study region (in the Swabian Jura), where *Taenia* species recovered their pretreatment prevalence level far earlier than *E. multilocularis* and *Mesocestoides* spp. after the cessation of treatment [29]. Compared with recent surveys of fox helminths from other parts of central Europe, it is obvious that the division into a high prevalence region of *E. multilocularis* in southern Germany and a low prevalence region in parts of northeastern Germany has remained stable for decades, as indicated by only 1.4% prevalence recently reported from foxes in the Uckermark [24]. This stability is reflected in Poland, where the division into high prevalence in the east and low prevalence in the west of the country has persisted since the early 2000s [25, 30].

The highly uneven frequency of *E. multilocularis* across Europe is contrasted by the remarkable uniformity of *Taenia* spp. frequency in foxes, where our prevalence data closely match recent data from northern Germany and various regions of Poland [24, 25]. This is unexplained and in need of study, but may be rooted in the different propagation strategies of *Taenia* with high reproductive potential in the definitive hosts, where even a small number of worms may be sufficient to infect the intermediate host community. Our prevalence data for *Mesocestoides* spp., although far higher than in the previous study in the same region [14], are on the low side compared with recent surveys from many other parts of Europe [25]. Also, between our three study sites, *Mesocestoides* showed the greatest variation of all examined helminths (30.2 to 69.3%). As neither the allocation to species is certain (although the largest part of the central European worms appears to belong to *Mesocestoides litteratus* [31]) nor the life cycle is fully known, these differences may be rooted in some aspect of the transmission system. Definitive hosts (e.g. foxes) acquire *Mesocestoides* by predation on rodents carrying tetrathyridia, but the infection route of rodents is yet unknown. It most probably involves some arthropod intermediate host, whose frequency may explain the different prevalence levels in definitive hosts. Our prevalence estimates for ascarid nematodes (we did not differentiate between *Toxocara* spp. and *Toxascaris leonina*) are in the range of frequencies reported from other parts of Europe [23–25]. Unlike with taeniid cestodes, there are various possible infection routes of *Toxocara* to foxes, but predation of rodents as paratenic hosts may play a more important role than previously assumed [32]. The common hookworm of foxes, *U. stenocephala*, is assumed to be mainly transmitted to the definitive host orally with soil containing L3 larvae, but, to an unknown extent, also through ingestion of rodents as paratenic hosts [33]. Prevalence in our areas was uniform, but rather moderate compared with surveys from elsewhere [23–25]. As studies on the infection mode (larvae from soil or paratenic hosts) or the impact of environmental parameters (e.g. soil moisture acting on the survival of larvae) are missing, no conclusions can be drawn. A minor factor could be reduced sensitivity of detection via the IST examination method. Infection of foxes with *A. alata* was low in two of our study areas and the parasite was completely absent in one (Ehingen/Donau). The patchy distribution and uneven frequency of this parasite are also known from elsewhere [25] and are assumed to reflect the dependency of the parasite’s life cycle on surface water bodies as the first stages parasitize planorbid snails and tadpoles of frogs. This parasite has become a matter of public health concern, as mesocercariae appear to be common in wild boar (*Sus scrofa*) as paratenic hosts. They are mostly present in the connective tissue of wild boar musculature, and may become transmitted to humans upon ingestion of uncooked boar products [34]. There are no human cases yet reported from Europe, but diagnosis of this parasite appears to be extremely challenging.

## Conclusions

We show that fox infection with *E. multilocularis* is evenly distributed within our sampling areas in a southern German high endemicity region for human AE. On small spatial scales, the distribution of human cases is heterogeneous, but fox infection is not. We conclude that other factors than the local intensity of the wildlife cycle are responsible for differences in human infection risk. We also show that the frequency of *E. multilocularis* in the study area has remained stable for the past 25 years, while other rodent- or soil-transmitted fox helminths, have increased in prevalence.

## Data Availability

The data supporting the findings of the study must be available within the article and/or its supplementary materials, or deposited in a publicly available database.
